# Evaluation of subacute subarachnoid haemorrhage detection using a magnetic resonance imaging sequence: Double inversion recovery

**DOI:** 10.37796/2211-8039.1058

**Published:** 2020-12-01

**Authors:** Zahra Mardanshahi, Maryam Tayebi, Sajad Shafiee, Maryam Barzin, Misagh Shafizad, Reza Alizadeh-Navaei, Abdolmajid Gholinataj

**Affiliations:** a Department of Radiology, Faculty of Medicine, Mazandaran University of Medical Sciences, Sari, Iran; b Auckland Bioengineering Institute, University of Auckland, Auckland, New Zealand; c Department of Neurosurgery, Faculty of Medicine, Mazandaran University of Medical Sciences, Sari, Iran; d Negin Medical Imaging Center, Sari, Iran; e Gastrointestinal Cancer Research Center, Mazandaran University of Medical Sciences, Sari, Iran; f Department of Anesthesiology, Faculty of Medicine, Mazandaran University of Medical Sciences, Sari, Iran

**Keywords:** Subarachnoid hemorrhage, Magnetic resonance imaging, MRI, Double inversion recovery

## Abstract

**Background and objectives:**

The diagnosis of subarachnoid hemorrhage (SAH) especially at the subacute stage is still a challenging issue using the conventional imaging modalities. Here we evaluated the role of double inversion recovery (DIR) sequence of MRI compared with the conventional gradient-recalled echo (GRE)-T2*-W and susceptibility-weighted imaging (SWI) sequences in the diagnosis of subacute SAH.

**Materials and methods:**

This prospective study was conducted on 21 patients with SAH, which were diagnosed using CT scan at the initial step. In the third week after the injury (14-20 days), all patients underwent a brain MRI exam that included T2*-W, SWI, and DIR imaging sequences. All images were independently read by two radiologists, who were blinded to the clinical history of the patients. The presence or absence of SAH was reviewed and assessed in 6 anatomical regions.

**Results:**

On the DIR images, 20 patients were found to have at least one subarachnoid signal abnormality, while the SWI and T2*-W images identified SAH areas on 17 and 15 patients, respectively. The highest rate of inter-observer consensus by the DIR sequence was found in the interhemispheric fissure and perimesencephalic area (*k* = 1). Also, a highest rate of inter-observer consensus using SWI was found in the interhemispheric fissure and posterior fossa cistern area (*k* = 1). A weak agreement was found in frontal-parietal convexity using SWI (*k* = 0.447), and in posterior fossa cistern by the T2* sequence (*k* = 0.447).

**Conclusion:**

In conclusion, the DIR sequence was more reliable at identifying signal abnormalities in subacute SAH patients than the T2*-W and SWI sequence, and is suggested as a promising imaging technique for detecting hemorrhagic areas without considering the anatomical distribution of SAH.

## 1. Introduction

Intracranial haemorrhage (ICH) as a common stroke subtype, caused by blood leaking into the brain parenchyma. This critical neurologic injury is responsible for 10-20% of strokes [1-5]. ICH comprises four different types: epidural and subdural hematoma, intracerebral haemorrhage, and subarachnoid haemorrhage (SAH). SAH arises from different conditions including traumatic (most common) and non-traumatic brain injury. Since SAH is a potentially life-threatening condition and may lead to disability, its accurate diagnosis and early management are of vital importance [6-10]. Delay or misdiagnosis of SAH occurs mostly in patients with headache presentation and even in patients undergoing a computed tomography (CT) scan. SAH may eventually lead to re-bleeding, further neurologic dysfunction, or even death [11-14]. Despite headache being one of the most important manifestations of this condition, up to 50% of SAH cases are misdiagnosed with a tension headache or migraine. This, in turn, is one of the reasons for delayed diagnosis and treatment, which can consequently be associated with unfavourable prognosis [15-17].

Methods for diagnosing SAH include patient presentation, lumbar puncture, brain imaging, and angiography. CT is highly sensitive at diagnosing SAH, and is thought of as a favourable imaging modality since it is relatively low-cost and efficient. The misdiagnosis rate for SAH via brain CT (performed within 6 hours of symptom initiation) was reported to be only 1.46/100,000 [18-21]. However, as more time passes from the onset of bleeding, the sensitivity of CT decreases due to loss of the density of haemorrhage on CT images [22]. As reported by Sames et al., by categorising SAH patients based on symptom onset time (less or more than 24 hours), the sensitivity of CT falls from 93.1% to 83.8% [23-24]. Furthermore, in the cases of minor bleeding and re-absorption of blood over time, CT seems unreliable and may provide false negative results [25-27].

Due to the aforementioned limitations of CT, Magnetic Resonance Imaging (MRI) has shown increasing use in the diagnosis and management of SAH. In particular, the Fluid-attenuated inversion recovery (FLAIR) sequence of MRI has shown superiority over CT in the diagnosis of SAH at both the acute and subacute phases [28]. Another conventional sequence of MRI, T2*-W imaging, has enabled one to discriminate between acute-subacute SAH from late-chronic bleeding phase by signal changes induced via iron oxidation [29]. However, in some cases, since MRI is prone to magnetic susceptibility artifacts, it can be difficult to detect subtle cases of SAH by FLAIR or T2*-W sequences [30]. Post-processing software that produce susceptibility weighted imaging (SWI) (based off T2*-W images) can also be used to identify patients subjected to SAH. As with T2*-W, the SWI sequence takes advantage of susceptibility differences between tissues and combines the magnitude and phase images to produce enhanced contrast magnitude images. The greater ability of SWI over T2*-W at detecting parenchymal and subarachnoid haemorrhage has been confirmed by several studies [31-32].

Another promising pulse sequence for the diagnosis of SAH is the double inversion recovery (DIR) sequence. DIR is an inversion recovery variant sequence which benefits from double non-selective 180 -inverting pulses and suppresses signal from both CSF and white matter simultaneously [33]. Since this sequence leads to clear delineation between sub-arachnoid spaces and the cerebral cortex, it can be considered to differentiate cortical lesions from SAH easily. Further, DIR sequence has been widely used for detection of multiple sclerosis plaques and cerebral cortex lesions because of its sensitivity to magnetic field variations [35-36]; however, to our knowledge, no studies except one, used this sequence in the detection of haemorrhagic areas in the brain. Therefore, the purpose of this study was to compare the DIR sequences compared with the conventional GRE-T2*-W and SWI sequence in detecting SAH.

## 2. Methods

This prospective study was conducted on patients with the definitive diagnosis of SAH who were admitted in the ICU department of Sari Imam Khomeini hospital. The patients were included in the study based on the clinical examination, which was done by a neurosurgeon, and CT scan at the hyper-acute stage (less than 10 hours after admission), reported by an expert neuroradiologist. Patients with hydrocephalus or those who had parenchymal haemorrhage close to SAH regions were excluded from the study. Furthermore, patients under 18 years old and those who had a contraindication to MRI (i.e. having MR-incompatible implanted devices, cardiac pacemaker, and claustrophobia) did not enter this project. Two weeks (14-21 days) after SAH diagnosis, MRI was performed for all selected patients in the subacute phase.

From May 2017 to June 2018, 25 patients who had all inclusion criteria with the diagnosis of SAH via unenhanced CT entered this study. Patients or their legal proxy filled out the consent form before considering in this study. Two patients were discharged before the MRI exam, and two of them were excluded due to poor MR image quality and motion artifact. From the 21 selected patients, seven had spontaneous SAH, 11 had sustained head trauma, and 3 had an aneurysm. There were 11 males and 10 females ranging from 20 to 62 years old with the mean age of 46.14 ± 11.27 years.

### 2.1. CT scan parameters

Axial CT scans of the brain were performed for all cases using a GE BrightSpeed 16-row detector scanner (GE Healthcare, Milwaukee, Wisconsin, USA): x-ray tube current = 140 mA, kVp = 120 kV, standard kernel, slice thickness = 5 mm, FOV = 220 mm.

### 2.2. MRI parameters

MR images were collected using a 1.5 T Aera, SIEMENS (Erlangen, Germany) and an 8-channel head coil with the following sequences: 2D T2*-W, 3D SWI, and 3D DIR. The total scan time was approximately 15 minutes for each patient. Different repetition (TR) and inversion (TI) values were tested in healthy volunteers to optimise the DIR sequence for the CSF and white matter suppression, simultaneously.

### 2.3. Image analysis

MR images were independently reported by two expert neuroradiologists (20 and 10 years of experience), who were blinded to the clinical data and CT scans of patients. Subarachnoid spaces of brain MR images were divided into six distinct anatomical regions: 1) frontal-parietal, 2) temporal-occipital, and 3) interhemispheric cisterns, 4) the Sylvian cistern, 5) perimesencephalic cisterns (both basal and mesencephalic cisterns), and 6) posterior fossa cisterns. After visually reviewing the images, the neuroradiologists were asked to give zero or one to each sub-arachnoid region in case of absence or presence of haemorrhage. To avoid recall bias, reviewers reported each set of images related to each sequence separately, in a randomised order, one week apart.

Diagnostic imaging criteria to detect SAH areas were as follows: high-attenuating, formless substances which filled the subarachnoid spaces around the brain in the axial non-enhanced CT images within less than 6 hours after symptom onset. In the subacute phase, on T2*-W images, SAH areas were found as low signal intensity in normally high SI subarachnoid spaces. The most dominant features in SWI images were the presence of hypointense SI in the sulci and cistern, which could be differentiated from veins by evaluating the smoothness and regularity of their shapes and uniformity of signals; such that the haemorrhagic regions were irregular compared with vessels, and had a rough boundary along with the non-uniform signal. On DIR images, hyperintense regions compared with the normal CSF were detected as SAH. After reviewing all images by the two neuroradiologists independently, they discussed their reports and came to an agreement. Then, the number of patients with at least one SAH location depicted by each MR sequence was counted based on each distinct anatomical area.

## 3. Statistical analysis

At the final stage, all statistical analyses were performed using SPSS.21 software (IBM Corporation, Armonk, USA). To find the level of interobserver agreement, Cohen's Kappa test was applied for each anatomical region and each sequence separately. Kappa values higher than 0.9 showed almost perfect agreement, between 0.9 and 0.8 strong, between 0.8 and 0.6, moderate, between 0.6 and 0.4, weak, between 0.4 and 0.2, minimal, and less than 0.2 suggested no agreement [37]. Note that the sensitivity and specificity of the sequences to depict SAH was not calculated since there was no standard criterion for subacute SAH validation. As such, we were not able to determine whether the additional areas found by each sequence was correct or not.

## 4. Results

A total number of 58 identified haemorrhagic areas were found using a combination of various MR sequences as presented in Table 1. The highest number of signal abnormalities found by DIR sequence alone was 29.31%. On the SWI and T2*-weighted images, 8 and 5 regions were identified, respectively. A consensus between all sequences was found in 9 areas (15.51%). Other common SAH locations found between two modalities were as follows: T2*-DIR 9, SWI –DIR 7, and T2*- SWI 3. As shown in Table 1, DIR was very accurate at detecting SAH in the posterior fossa cistern (6 out of 6), frontal-parietal areas (14 out of 16), and Sylvian cistern (5 out of 6). Most of the subarachnoid bleeding in temporal-occipital regions were identified by the T2*-w sequence (8 out of 13). SWI outperformed the other sequences in the interhemispheric fissure, detecting 6 out of 9 SAH regions. All sequences were almost equally sensitive at detecting SAH in the perimesencephalic cistern.

**Fig. 1:**
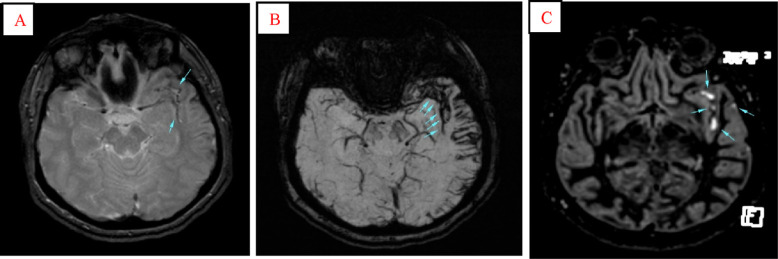
Spontaneous subarachnoid haemorrhage in a 44-year-old woman. MR images were performed 18 days after injury. Using T2*-W (A, arrows) and SWI (B, arrows) images, detection of SAH is a challenging issue due to its adjacency to main veins in the base of the skull and the similarity of signal intensity between these veins and haemorrhage may lead to misdiagnosis of SAH in this region of brain. In DIR image (C, arrows), SAH is demonstrated as marked several hyper-intense regions in the left Sylvian cistern. Note that detecting SAH in this area is one the priorities of DIR sequence to T2* and SWI.

**Fig. 2:**
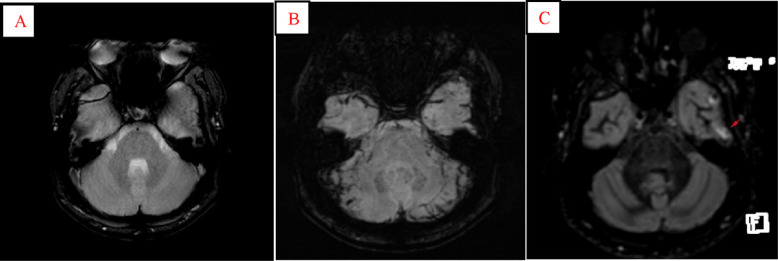
SAH in temporooccipital subarachnoid space in a 32-year-old man who done MRI 17 days after traumatic injury. In axial T2* (A) and SWI (B) images, no signal abnormality is detected because of the destructive susceptibility artifact from air-tissue interfaces in the temporal lobes. Conversely, distinguished signal intensity in DIR (C, arrow) image is observed.

**Fig. 3:**
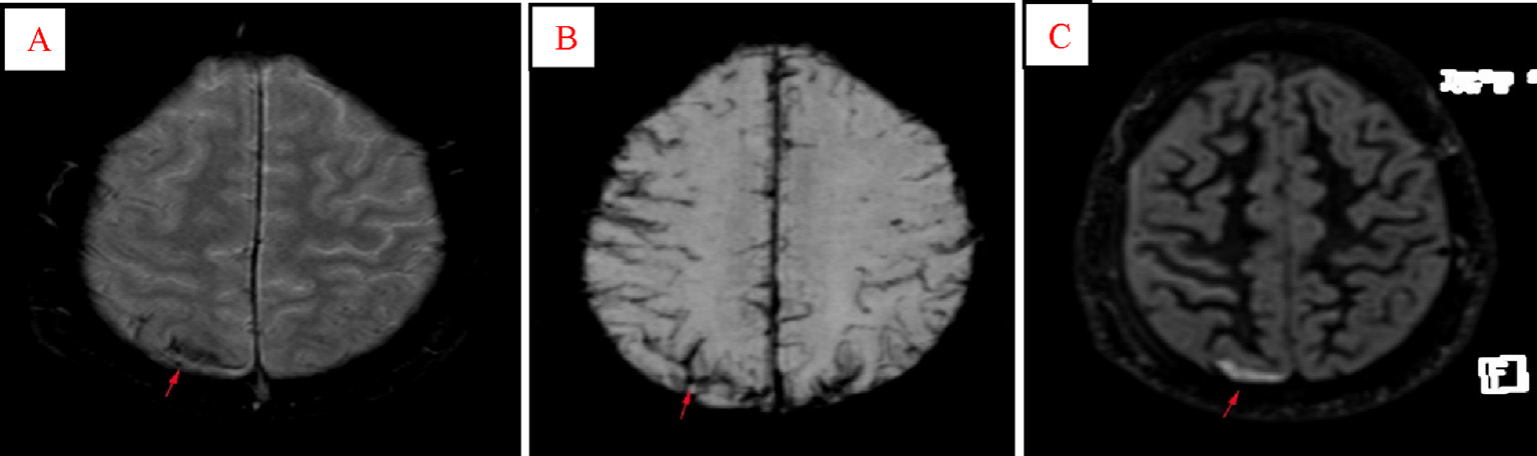
Fronto-parietal SAH in a 21-year-old man with traumatic brain injury. Haemorrhagic regions in T2*-W (A, arrow) is less visible than SWI (B, arrow), whereas DIR (C, arrow) showed SAH as a significant hyper-intense location in the right parietal sulcus.

The highest rate of inter-observer consensus was found by the DIR sequence in the interhemispheric fissure, the perimesencephalic and posterior fossa cistern, and the frontal-parietal sulci (p-value<0.0001). Perfect agreement was also found between readers using SWI sequence in the interhemispheric fissure and posterior fossa cistern and using T2*-W in the frontal-parietal areas (p-value<0.0001). Weak agreement was found in temporal-occipital (p-value = 0.004) and frontal-parietal convexity (p-value = 0.019) using SWI, as well as in Sylvian cistern (p-value<0.0001) and posterior fossa by the T2*-W sequence (p-value = 0.018) (Table 2 and Figs. 1–3).

Among 21 patients, the DIR sequence found 20 patients with at least one subarachnoid signal abnormality, and SWI and T2*-w sequences identified SAH areas on 17 and 15 patients, respectively (Table 3).

## 5. Discussion

This study indicated that DIR is highly sensitive to microhaemorrhage compared with T2*-W and SWI sequences at the subacute phase of sub-arachnoid haemorrhage. Additionally, DIR has a high potential to detect subtle SAH in all sub-arachnoid spaces compared with T2*-W and SWI. At the subacute phase, the T2*-W sequence is more sensitive to small regions of haemorrhage in subarachnoid spaces because of the paramagnetic effects of haemoglobin products in this time course [38]. This effect leads to the loss of signal intensity and causes SAH areas to appear as dark regions on these series of images. The pitfall of this sequence is the presence of magnetic susceptibility between the skull and brain which leads to a ‘blooming’ artifact and missing SAH regions at the skull base [39].

SWI has similar issues as the T2*-W sequence; however, it is more sensitive to the high concentration of iron products of haemorrhage in subarachnoid spaces. The 3D SWI, as a high-resolution sequence, suffers less from the partial volume effect and can detect microhaemorrhages especially in the inter-hemispheric fissure, supracerebellar cistern, and interventricular areas. On the contrary, a lower detection rate of SAH was observed in the temporal-occipital convexity and Sylvian cistern which might be due to the susceptibility artifact resulting from adjacency to air-tissue interface [40-41] Consistent with the Verma et al. [23], the results of the present study revealed that the SWI alone identified the maximum number of SAH regions in the interhemispheric areas, while no abnormal area was found in the Sylvian cistern. Furthermore, none of the SWI and T2*-W sequences detected haemorrhage in the posterior fossa, which can be elucidated by their limitation at finding SAH in the base of the skull.

Paramagnetic products can cause an aliasing artifact, which is destructive for MR images. In the phase image of SWI sequence, Wu et al. found that most of the SAH areas are along with this artifact, while veins are not aliased on these series of images. So, they concluded that the aliasing artifact could be considered as a distinguishable factor [32]. However, several confounding factors can also cause an aliasing artifact, including air-filled spaces, calcification, and fast flow from the middle cerebral artery. Thus, it is difficult to differentiate hypointense cortical veins from SAH on SWI images. In line with Hodel et al. [41] our findings also demonstrated a lower number of SAH identified by SWI and T2*-W compared with the DIR sequence in the periphery and convexities of the brain. This difference can arise from the similarity in signal intensity between the paramagnetic effects of deoxyhaemoglobin in the cortical veins and methaemoglobin in the sub-arachnoid haemorrhage regions appearing as hypointense areas in these series of images. So, this issue can clarify the lower inter-observer consistency rate in T2*-W and SWI than the DIR sequence. Furthermore, this sequence could identify almost all patients with SAH (20 out of 21) and more than 70% of haemorrhagic regions. In the present study, applying a three-dimensional DIR sequence with thin slice thickness improved the SNR and spatial resolution along with a reduction in partial volume effect, thereby enhancing detection of SAH areas. Further, this sequence is less prone to susceptibility artifact than the T2*-W and SWI approaches, making it more authentic for identifying suspected SAH regions in the posterior cranial fossa [34]. We postulate that the higher SNR and fewer confounding artifacts of DIR can give rise to a strong agreement between readers. In this study, the CT scan was only performed for patients at the hyper-acute phase of haemorrhage, so it was not applicable to compare CT findings with other MRI sequences at the subacute stage. Additionally, in many studies, it has been shown that the sensitivity of CT to detect aneurysmal SAH decreases over time from around 92% in the first 24 hours to less than 50% after one week [32]. This significant decline in SAH detection is directly related to the reduction in haemoglobin concentration, because of protein reabsorption and CSF circulation which redistributes focal SAH [38]. On the other hand, CT is not capable of finding subtle haemorrhages in the posterior fossa, due to beam hardening artifacts and higher partial volume effect [40].

In this study, we had several limitations which should be resolved for future studies. First, we did not recruit healthy volunteers to compare their MR images with SAH patients. Second, there were no standard criteria to definitely determine the hypo-or hyper-intense regions found in different sequences as SAH. Thus, while the DIR sequence was able to detect more signal abnormalities than other sequences, there was no standard reference to prove the nature of those abnormalities. Performing an additional CT scan for the patient in the subacute phase is not a routine diagnostic procedure in our hospital, so based on ethical considerations and financial issues, we were not able to compare our results with CT images, as performed by previous studies. Finally, most of the qualified patients for this study were hospitalised in the ICU department, and were not at a reliable level of consciousness to undergo MR imaging at the right time, limiting our sample size to a relatively small number of patients.

## 6. Conclusion

Overall, MRI-based DIR images have a higher ability to detect and delineate signal abnormalities than T2* and SWI images in SAH patients who are in the subacute phase of injury. It was also observed that the diagnostic values of SWI and T2*-W sequences are strongly associated with the anatomical distribution of SAH. Hence, the DIR sequence is suggested as a promising imaging technique to find haemorrhagic areas in all brain regions of patients several days post-injury.

## Figures and Tables

**Table 1:** Number of haemorrhagic areas (SI abnormality) detected in the various regions of subarachnoid space by different MR sequences after readers’ consensus.

Fronta-parietal	Temporal- occipital	Interhemispheric fissure	Sylvian cistern	Perimesencephalic cistern	Posterior fossa cistern	Total	Fronta-parietal
T2*-W only	0	2	1	0	2	0	5
SWI only	2	1	4	0	1	0	8
DIR only	4	2	2	2	3	4	17
T2*-W and SWI	0	2	0	1	0	0	3
T2*-W and DIR	5	3	0	1	0	0	9
SWI and DIR	2	2	0	1	1	1	7
T2*-W, SWI and DIR	3	1	2	1	1	1	9
Total	16	13	9	6	8	6	58

**Table 2:** Inter-observer agreement for all applied MR sequences between 2 neuroradiologists based on various anatomical areas.

	T2* -W (95% CI)	SWI (95% CI)	DIR (95% CI)
Frontal-parietal	1.000 (1-1)	0.447 (0.030 –0.731)	0.882 (0.733-0.951)
Temporal- Occipital	0.679 (0.360 –0.856)	0.551 (0.168 -0.790)	0.716 (0.420-0.874)
Interhemispheric fissure	0.716 (0.421 –0.874)	1.000 (1-1)	1.000 (1-1)
Sylvian cistern	0.542 (0.155 –0.785)	0.798 (0.566-0.913)	0.816 (0.599-0.921)
Perimesencephalic cistern	0.783 (0.538 –0.906)	0.783 (0.538 –0.906)	1.000 (1-1)
Posterior fossa cistern	0.447 (0.030 –0.732)	1.000 (1-1)	0.882 (0.733-0.951)

**Table 3:** Number of patients with at least 1 SAH area found for different MR sequences after an agreement between readers.

	T2*-W	SWI	DIR
Frontal-parietal	8	6	12
Temporal- Occipital	4	3	8
Interhemispheric fissure	4	4	4
Sylvian cistern	2	3	4
Perimesencephalic cistern	2	2	4
Posterior fossa cistern	1	1	5
Total number of patients	15	17	20
